# Post-interaction neuroplasticity of inter-brain networks underlies the development of social relationship

**DOI:** 10.1016/j.isci.2024.108796

**Published:** 2024-01-04

**Authors:** Simone G. Shamay-Tsoory, Inbar Z. Marton-Alper, Andrey Markus

**Affiliations:** 1 Department of Psychology, University of Haifa, Haifa, Israel; 2 The Integrated Brain and Behavior Research Center (IBBRC), Haifa, Israel

**Keywords:** Behavioral neuroscience, Cognitive neuroscience

## Abstract

Inter-brain coupling has been increasingly recognized for its role in supporting connectedness during social communication. Here we investigate whether inter-brain coupling is plastic and persists beyond the offset of social interaction, facilitating the emergence of social closeness. Dyads were concurrently scanned using functional near infrared spectroscopy (fNIRS) while engaging in a task that involved movement synchronization. To assess post-interaction neuroplasticity, participants performed a baseline condition with no interaction before and after the interaction. The results reveal heightened inter-brain coupling in neural networks comprising the inferior frontal gyrus (IFG) and dorsomedial prefrontal cortex in the post-task compared to the pre-task baseline. Critically, the right IFG emerged as a highly connected hub, with post-task inter-brain coupling in this region predicting the levels of motivation to connect socially. We suggest that post-interactions inter-brain coupling may reflect consolidation of socially related cues, underscoring the role of inter-brain plasticity in fundamental aspects of relationship development.

## Highlights

• Inter-brain coupling increases following social interaction offset
• We found higher inter-brain coupling post-interaction compared to pre-interaction
• Post-task inferior frontal inter-brain coupling predicted social connectedness levels
• Inter-brain plasticity is a core aspect of relationship development

## Graphical abstract

**Figure:**
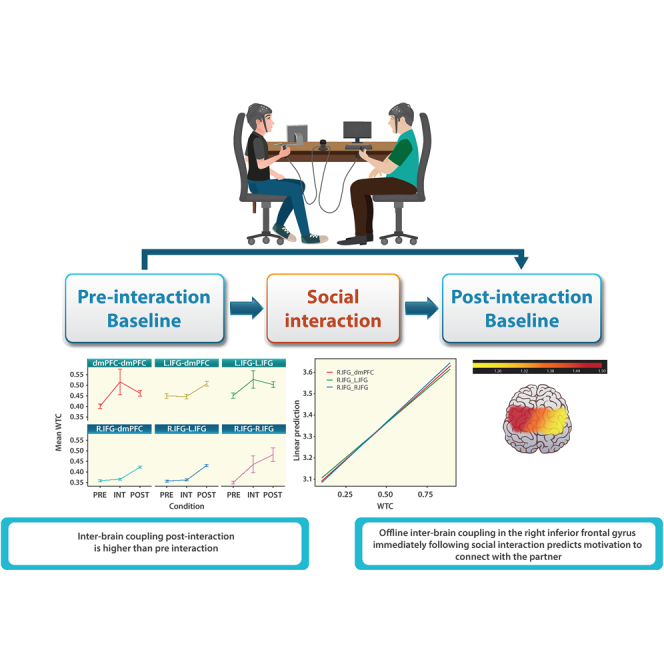

## Introduction

During social encounters, interaction partners transfer personal and general information that may contribute to the development of social relationships. Recent attempts have been made to understand the formation of social interactions in real-life settings with methods allowing the simultaneous assessment of brain and behavior.^1^^,^^2^ This growing field has offered new evidence showing that functional connectivity between brain regions of two interacting individuals underlies various types of interactions.^2^

Specifically, previous studies have reported inter-brain coupling in regions associated with mirroring and mentalizing processes during tasks that involve coordinated actions, such as synchronized walking^3^ or rhythmic joint movement.^4^ For instance, the inferior frontal gyrus (IFG), which is a crucial region within the observation-execution system,^5^ playing a role in comprehending actions by representing the motor commands necessary to perform those actions,^6^ was consistently observed to exhibit inter-brain coupling in dyads and groups during social interactions.^7^ Inter-brain coupling in the IFG was reported also in tasks involving verbal coordination including cooperative song learning,^8^ dyadic dialogue,^9^ joint singing^10^ and verbal discussions^11^^,^^12^ as well as non-verbal communication, such as fingertip moving^13^ and playing Jenga game.^12^^,^^14^

Furthermore, the mentalizing network, which is involved in processes related to understanding the thoughts and intentions of others, has also been found to exhibit inter-brain coupling during social interactions. Specifically, the dorsomedial prefrontal cortex (dmPFC), a critical region for mentalizing and differentiating between self-related and other-related representations,^15^^,^^16^^,^^17^ has been shown to display coupled activity between two brains during cooperative tasks.^12^ A recent study examining coupling between dmPFC neurons of pairs of mice identified coupling in neuronal activity between the two animals during social interactions,^18^ indicating that this region may code mutual predictions during interactions.

However, it remains uncertain whether inter-brain coupling, which emerges during social interactions, undergoes dynamic changes following the interactions. In theory, interacting with a stranger for the first time requires integration and registration of the bidirectional information cues communicated by the interaction partners. It could be the case that brain regions activated during the interaction continue to be activated following the offset of the interaction in the aim of registering interaction related cues into memory for the purpose of creating social relationships. These cues may include the movement rhythms, prosody, emotions, or verbal responses of the interaction partner. While traditional memory studies have centered on memory-related activations during task encoding,^19^ it is increasingly acknowledged that brain activations that occur *following* encoding may predict follow-up outcomes.^20^ Indeed, a growing body of research on neuroplasticity across diverse domains has provided evidence that post-task changes in brain activity and connectivity are reflective of recent cognitive experiences.^21^ These dynamic changes in brain activity have been shown to contribute to subsequent memory performance and learning.^22^^,^^23^ Zhang et al.^24^ argued that post-task activations represent covert attempts to rehearse a recently completed task, a process which is hypothesized to reengage the recent task-dependent brain connectivity.

It is therefore plausible to suggest that post-interaction inter-brain coupling serves as a mechanism for reengaging with interaction-related information, including the sense of mutual closeness and connectedness. This view is supported by behavioral findings indicating that during tasks involving movement synchronization, interaction partners tend to maintain synchronization even after the interaction has ended.^25^ For example, dyads walking synchronously were shown to continue walking at the same pace even after they separate.^26^ Oullier et al.^27^ suggested that this form of carry-over effect of synchronization may allow the development of social memories of partners engaged in social interaction. Notably, a recent electroencephalogram (EEG) study revealed post-interaction increase in inter-brain coupling in delta band following joint music playing.^28^ As inter-brain plasticity was suggested to underlie interaction-based learning,^29^ it is possible that post interaction inter-brain coupling may serve as a potential mechanism for the development of social relationships. Indeed, it was found that following synchronization, interaction partners show increased cooperation,^30^ increased affiliation,^27^^,^^31^ cohesion,^32^ and connectedness.^33^ Furthermore, it was revealed that that the outcome of first romantic dates depends on the level of electrodermal synchrony and attunement of behavior,^34^ indicating that synchronization may have emotional carry over consequences. Finally, Koul et al.^35^ have recently demonstrated with Granger causality analysis, that synchronized social behaviors cause inter-brain coupling, such that synchronized movement leads to inter-brain coupling^35^ which may persist over time.

**Figure 1:**
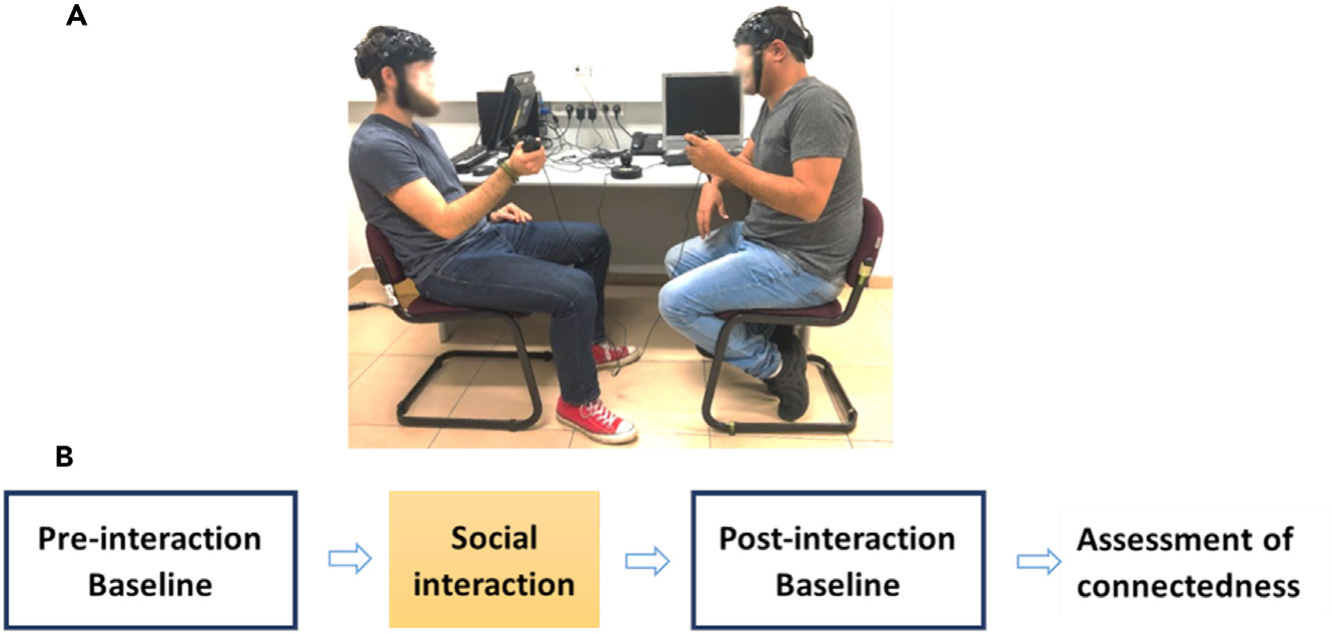
Paradigm design (A) To induce face-to-face social interaction (INT), we utilized a movement synchronization paradigm. Participants were directed to hold a three-dimensional game controller with one hand and perform synchronized movements, including coordinated actions such as moving right, left, forward, backward, in circles, and similar motions. (B) Baseline assessments of inter-brain coupling were conducted immediately before (PRE) and after (POST) the social interaction.

**Figure 2:**
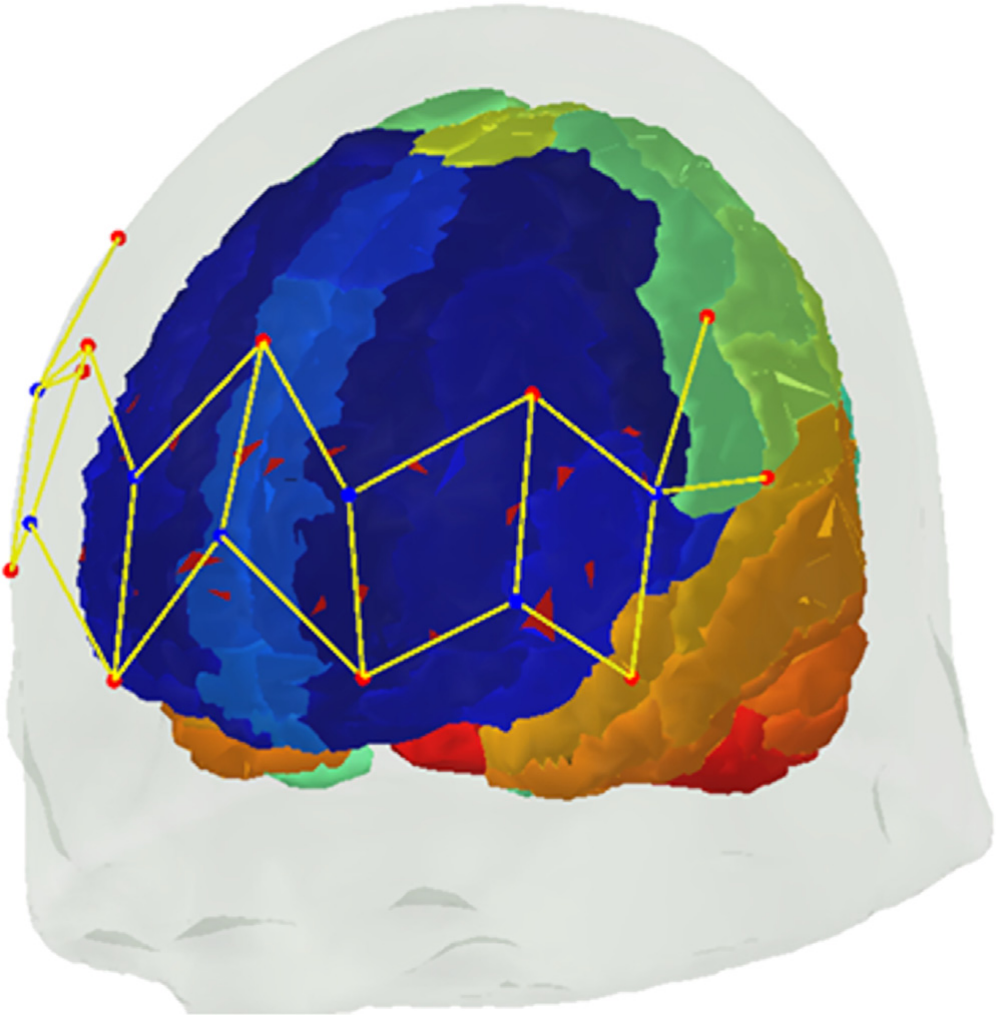
fNIRS channel placement against anatomical brain areas Channel (marked as yellow lines) are formed between transmitters (red dots), and adjacent receivers (blue dots).

Collectively, the persistence of inter-brain coupling following interactions may point to some consolidation of social cues that occurs following social interactions. The interaction partners’ movement representation may continue to be active in memory even when the exchange of social cues is discontinued,^36^^,^^37^ which serves to promote future relationship. To investigate post task inter-brain coupling, we analyzed data of a social interaction paradigm involving movement synchronization of 32 dyads,^38^ with pre- and post-interaction baseline conditions consisting of back-to-back movement (Figure 1). Assessing task related inter-brain coupling allowed us to identify the brain regions exhibiting increased post-interaction coupling. The functional near infrared spectroscopy (fNIRS) channels covered the IFG and dmPFC (Figure 2) and we focused on inter-brain coupling in the IFG and dmPFC which can occur between homologous regions (such as IFG-IFG) but also between non-homologous regions (like IFG-dmPFC). In complex social interactions, it is anticipated that both types of coupling will take place. For instance, in a conversation involving listening and speaking, brain areas responsible for language processing and comprehension in one individual might exhibit coupling with brain areas responsible for speech production and expression in another individual, forming coupling between homologous and non-homologous regions. Concerning the coupling observed in the IFG and dmPFC, our prediction was that regions associated with movement observation-execution and mentalization would be coupled both within and between inter-brain networks. Critically, we thus hypothesized that brain regions engaged in the generation of movement synchronization including the IFG and dmPFC, continue to be coupled following the termination of the interaction. Finally, building on studies showing that movement synchronization generates connectedness,^32^ we predicted that post-interaction inter-brain coupling would predict follow-up levels of motivation to connect assessed in a self-report scale.

## Results

### Real vs. pseudo pairing

**Figure 3:**
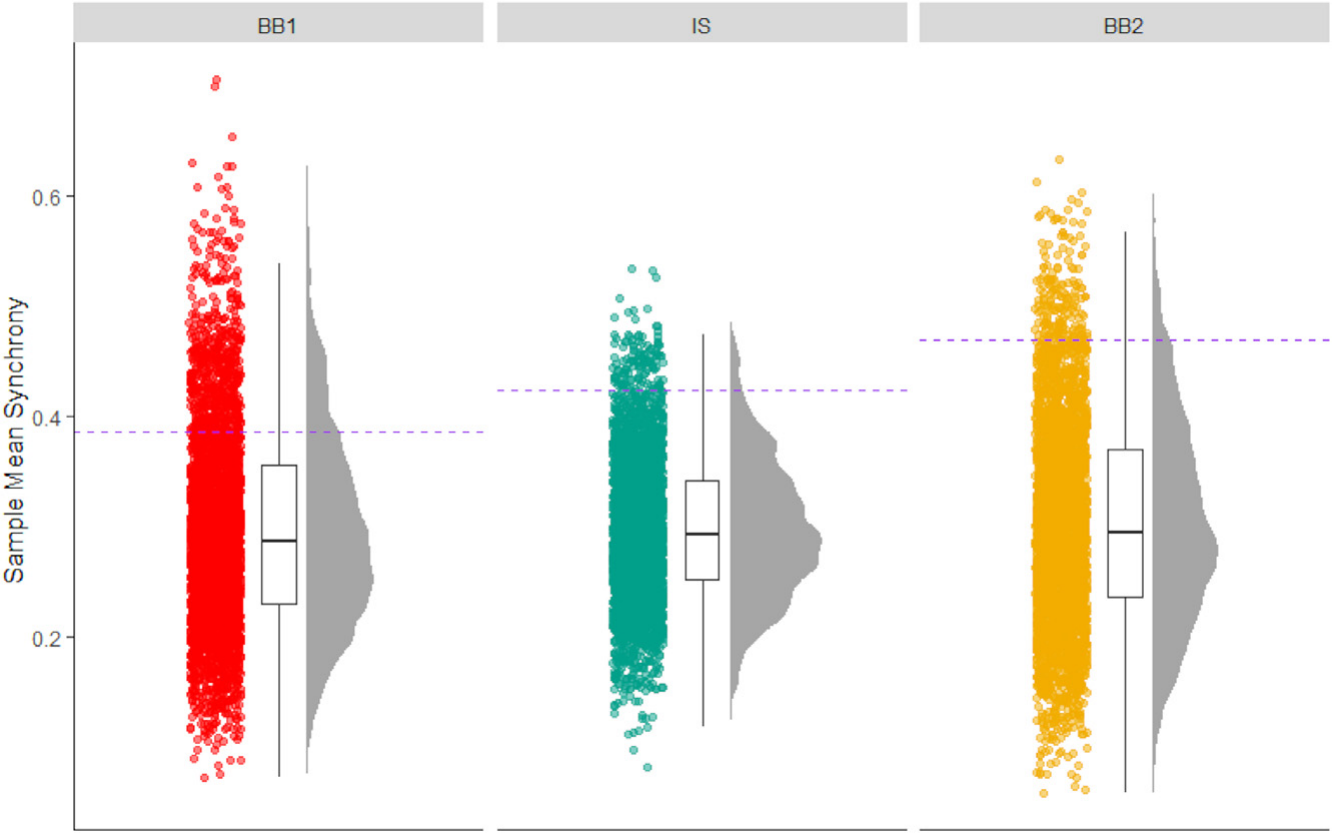
Inter-brain coupling in the INT condition in the true sample compared to random permutations Distribution of coupling in dyads 2,919 samples generated by randomly pairing meetings. The dashed line represents the mean of inter-brain coupling in the true sample.

fNIRS data collected from the participants during the task were pre-processed using the HOMER2^39^ package for the MATLAB programming language. Following the findings of Hoshi,^40^ we chose the O_2_Hb values for further analyses, as more representative of cerebral blood flow. Coupling between regions within and between brains was estimated using a Wavelet Transform (WTC) function.^41^ To test whether inter-brain coupling in the sample was above chance, we created 2919 pseudo-samples by randomly pairing dyads that did not interact with each other (Figure 3). Mean inter-brain coupling in the true sample in the INT condition (*m*_*(SD)*_ = 0.424 (0.798)) was outside of a 95% confidence interval of randomly permuted samples (95% CI 0.441). No dyad of the 2919 permutations had higher mean coupling than the true sample. On the other hand, the mean of inter-brain coupling in the PRE condition (*m*_*(SD)*_ = 0.385(0.282)) was within the 95% confidence interval of randomly permuted samples (95% CI 0.5), while in the POST condition the mean inter-brain coupling was marginally within the range (*m*_*(SD)*_ = 0.469 (0.469), 95% CI 0.507).

### Differences between conditions in inter-brain coupling

We used Linear Mixed Effects (LME) models using the R language,^42^ and the *lme4* package for the R language.^43^ The LME model included Condition (PRE, POST, INT), and ROI pairings as fixed factors, dyads’ ID numbers as a random factor, and coherence (WTC) values as a dependent measure. The first model included only the main effects of the fixed factors, while the second model included a possible interaction between them as well. Type II Wald χ^2^ comparison tests showed that the second model yielded a significantly greater predictive power than the first model [χ^2^_(10)_ = 27.69; p < 0.005], and was therefore used in further analyses. Analysis of the second model revealed a significant main effect of ROI F_(5, 10994)_ = 17.06, p < 0.0001, η_p_^2^ = 0.0077], main effect of condition F_(2, 10993)_ = 20.25, p < 0.0001, η_p_^2^ = 0.0037], with overall, higher inter-brain coupling in the INT condition compared to Pre condition [t_(10993)_ = 4.79; p < 0.0001] and higher inter-brain coupling in the Post compared to Pre condition [t_(10993)_ = 6.03; p < 0.001]. Importantly we found a significant interaction [F_(10, 10993)_ = 2.76, p < 0.01, η_p_^2^ = 0.0025] between Condition and ROI such that in the R.IFG- R.IFG ROI coherence in the Pre condition (M = 0.347, SD = 0.157) was significantly lower than that in the INT (M = 0.494, SD = 1.485) [t_(10993)_ = 3.85; p < 0.001] and the Post condition (M = 0.512, SD = 1.07) [t_(10993)_ = 4.44; p < 0.001], with no significant difference between the INT and Post conditions [t_(10993)_ = 0.56; n.s.]. In the R.IFG-dmPFC ROI coherence in the Pre condition (M = 0.347, SD = 0.144) was significantly lower than that in the Post (M = 0.43, SD = 0.179) [t_(10993)_ = 1.62; p < 0.01], with no significant difference between the PRE and IS conditions [t_(10993)_ = 0.79; n.s.] and INT and Post condition [t_(10993)_ = 2.17; n.s.].

In the dmPFC-dmPFC ROI the coherence in the Pre condition (M = 0.392, SD = 0.251) was significantly lower than that in the INT (M = 0.553, SD = 1.669) [t_(10993)_ = 3.25; p < 0.01], with no significant difference between the Pre and Post conditions [t_(10993)_ = 1.95; n.s.] and INT and Post condition [t_(10993)_ = 2.17; n.s.]. In the LIFG-LIFG ROI, the coherence in the Pre condition (M = 0.453, SD = 0.441) was significantly lower than that in the INT (M = 0.547, SD = 1.402) [t_(10993)_ = 2.46; p < 0.01], with no significant difference between the Pre and Post conditions [t_(10993)_ = 1.63; n.s.] and INT and Post condition [t_(10993)_ = 0.834; n.s.].

In the LIFG-RIFG ROI, the coherence in the Pre condition (M = 0.351, SD = 0.155) was significantly lower than that in the Post (M = 0.438, SD = 0.195) [t_(10993)_ = 3.42; p < 0.01]. Similarly, the INT (M = 0.368, SD = 0.149) condition was significantly lower than the Post condition (M = 0.438, SD = 0.195) [t_(10993)_ = 2.75; p < 0.05], with no significant difference between the PRE and INT conditions [t_(10993)_ = 0.67; n.s.].

In the LIFG-dmPFC ROI, the coherence in the INT condition (M = 0.423, SD = 0.332) was significantly lower than that in the Post (M = 0.497, SD = 0.366) [t_(10993)_ = 2.56; p < 0.01], with no significant difference between the PRE and INT conditions [t_(10993)_ = 0.6; n.s.] and Pre and Post condition [t_(10993)_ = 1.96; n.s.].

**Figure 4:**
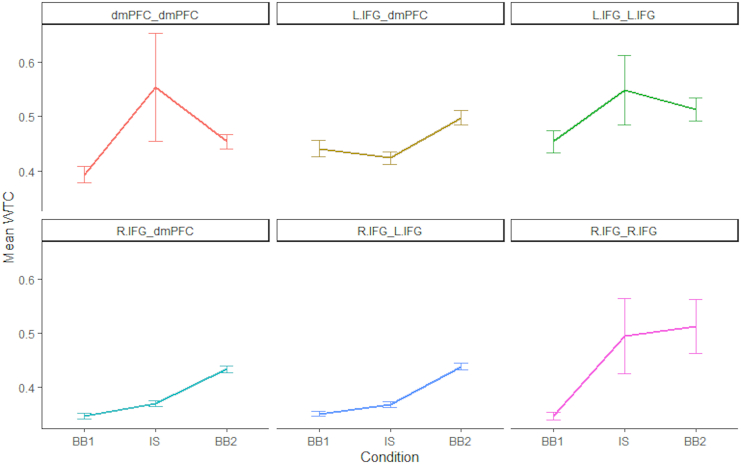
Inter-brain levels between in the PRE, INT, and POST conditions Changes in WTC in the 6 ROIs.

To conclude, in three ROIs (rIFG-rIFG, rIFG-lIFG, and rIFG-dmPFC) we found a significant difference between the Pre and the Post conditions. The same pattern of differences between the Pre and Post and Pre and INT was observed in the RIFG-RIFG, indicating carryover effect of inter-brain coupling in this region (Figure 4).

### Inter-brain coupling as a predictor of motivation to connect

**Figure 5:**
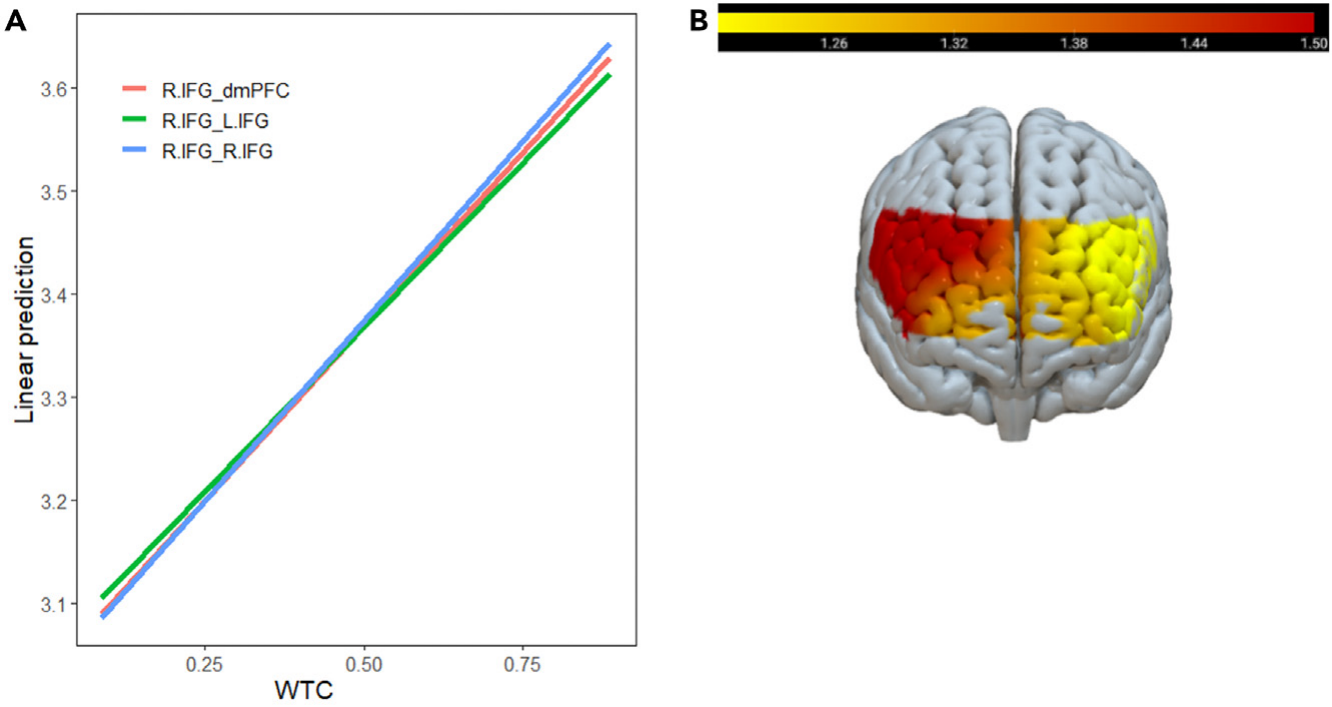
Inter-brain coupling in the RIFG predicts motivation to connect (A) Inter-brain coherence in the R.IFG-R.IFG, R.IFG-L.IFG, and dmPFC-R.IFG, showing significant prediction of motivation to connect. (B) Prediction magnitudes of motivation to connect by inter-brain coupling, in the R.IFG-R.IFG, R.IFG-L.IFG, and dmPFC-R.IFG. Color denotes the slope of the linear prediction.

We further examined the predictive value of inter-brain coupling between brain regions for the motivation to connect. The motivation to connect was assessed at the end of the experiment with a scale asking about the extent to which they wish to talk with the interaction partner in the future. In this analysis, we used a subset of the three ROIs (R.IFG-R.IFG, R.IFG-dmPFC, and L.IFG-R.IFG) that showed significant difference between Pre and Post conditions, and calculated WTC values within each participant’s brain. We constructed three LME models, each consisting of Condition (Pre/Post), ROI, and the continuous value of WTC as fixed factors, and participant number as a random factor. The models differed in the level of interaction between the three fixed effects, such that the first model included only the main effects of the fixed factors, the second model additionally included all 2-way interactions between the fixed factors, and the third model included main effects and all possible interactions between the fixed factors. A Type II Wald χ2 test showed that the second model provided a significantly better prediction compared to the first model [χ^2^_(5)_ = 52.24; p < 0.0001], whereas the third model did not provide a better prediction than the second model [χ^2^_(2)_ = 3.27; n.s]. Examination of the model, which included all interactions between Condition, ROI, and WTC, was used in further analyses in this section. Examination of the this model yielded a significant interaction [F_(1,5253)_ = 50.154, p < 0.0001, η_p_^2^ = 0.01] between Condition, and WTC coherence. Results revealed that in the Post condition, in the R.IFG- R.IFG ROI, WTC coherence was a significant positive predictor of motivation to connect [t_(5248)_ = 2.28; p < 0.05]. Similar effects were observed in the R.IFG- L.IFG [t_(5248)_ = 2.85; p < 0.01] and in the R.IFG-dmPFC IFG [t_(5248)_ = 2.61; p < 0.01]. A summary of brain and behavior relationship are presented in Figure 5 which depicts the association between and inter-brain coupling in the R.IFG post intereaction and motiation to connect.

## Discussion

Here we investigated whether inter-brain coupling following the offset of a short social interaction is increased, compared to baseline coupling before the interaction. Our paradigm allowed us to distinguish between inter-brain coupling before, during, and following an interaction. As we were interested in understanding how inter-brain coupling following the interaction predicts the development of social relationships, we probed levels of motivation to connect following the interaction.

To ensure that our inter-brain coupling measures reflect real social interaction, we initially compared inter-brain coupling in real dyads to that in pseudo dyads, obtained by premutation of coupling of participants from real dyads. The results of this analysis replicated previous reports showing that overall inter-brain coupling during real social interaction is higher than in pseudo-paired participants. These findings further confirm that inter-brain coupling is not an epiphenomenon of doing the same thing at the same time and validate inter-brain coupling as a compelling measure representing the emergence of between-brain network occurring during real face-to-face social interaction.

When comparing inter-brain coupling before, during, and after the social interaction, we found that inter-brain coupling during the social interaction condition was significantly higher than pre-interaction inter-brain coupling between the R.IFG-R.IFG, L.IFG-L.IFG, and R.IFG-dmPFC and dmPFC-dmPFC. The involvement of the IFG and the dmPFC in face-to-face synchronized social interaction is in accordance with our hypothesis that the observation-execution and the mentalizing systems support the emergence of social alignment during social interactions. The dmPFC is also part of a system that tracks gaps between self and others and responds to misalignment.^44^ Evidence from studies on social alignment reveals that this region is activated during social deviations,^45^^,^^46^ indicating that this region supports the detection of gaps between the self and others with the aim of promoting realignment.

While inter-brain coupling increased during social interactions, we found that in several regions post-interaction inter-brain coupling was higher than pre-interaction inter-brain coupling. Specifically, post-interaction inter-brain coupling was significantly higher than pre-interaction coupling between the rIFG-rIFG, rIFG-lIFG, and rIFG-dmPFC. Moreover, analyses of brain and behavior relationships indicated that post-interaction inter-brain coupling in these regions predicted self-reported levels of motivation to connect with the other person in the future. This demonstrates that increased post-interaction inter-brain coupling may represent preparedness for future relationship. It is possible that the correlation analysis between the motivation to connect scale and inter-brain coupling is indictive of inter-brain coupling being a predictor of the level of social connectedness. This indicates that carry over effects of inter-brain coupling are negligible in cases of low social connectedness.

Notably, the right IFG was found as a common denominator in all inter-brain coupling combinations, indicating that this region is a major hub of brain networks organization during the emergence of social relationships. Previous neuroimaging studies reveal that the right IFG supports imitation,^47^ coordination,^48^ coding of intentions^49^ and perception-action matching.^50^ A recent meta-analysis by^2^ found that different subregions of the PFC, particularly the IFG, show inter-brain coupling during various joint games involving cooperation.^10^^,^^12^^,^^15^ Recent research conducted by Wei et al.^51^ has demonstrated that enhanced inter-brain coupling in the right IFG during cooperative tasks correlates with superior task performance. In our paradigm, during the interaction condition, synchronization involved movement coordination. It was suggested that during synchronization of movements interaction partners have to extract temporal structures from the other movements, generate an internal model of rhythm patterns, and predict their next movement.^52^ As the right IFG is part of a system that supports structure prediction of rhythms^47^, it may be argued that this region has general relevance for coding rhythmic patterns during social interactions. Here, we complement this picture by providing evidence that inter-brain coupling in the right IFG continues following an interaction, indicating that it may continue to code rhythmic patterns following the interaction’s offset.

How might the post-interaction inter-brain coupling contribute to the formation of social relationships? Considering that results from studies examining post-event cognitive responses occurring minutes (to hours) following the exposure to the event are interpreted as reflecting system consolidation,^53^^,^^54^^,^^55^ the current findings too can be viewed as reflecting consolidation of socially related cues. Connectedness, in this sense, requires binding of information from various emotional and social cues to create a cohesive representation of the interaction as a whole. This process allows individuals to integrate and make sense of the different elements present in social interactions, such as emotions, facial expressions, body language, vocal intonations, and verbal content. This idea is in line with early studies on consolidation in rodents, which reveal that during periods of rest or sleep, hippocampal neurons replay sequences of rhythmic firing that were recorded during learning.^56^^,^^57^^,^^58^ If the right IFG codes the structure of movement rhythms, the replay of activity may include a coupled replay of sequences of movements by the participants. The carry over effects of inter-brain coupling found here, may thus reflect some sort of replay of interactive movement rhythm structures to create stable representation of the interaction for future encounters with the same social agent. Accepted theories of neuroplasticity, such as the spike-timing-dependent plasticity principle,^59^ hold that when two brain regions discharge proximately in time, connections between these regions will be enhanced. The inter-brain plasticity approach expands the spike-timing-dependent plasticity principle, proposing that when two brain regions, in different brains, are activated simultaneously the coupling between them increases.^29^ Replay of the activity in the regions that were activated during interaction in the same rhythms may underlie the future outcome of the relationship. It is thus possible that post-interaction activity reflects an offline consolidation process whereby interaction related-traces develop into stable representations and are registered into memory. Thus, the extent of the post-interaction coupling may reflect the extent of the consolidation, which would imply that the consolidation occurs during this post-interaction activity.

### Limitations of the study

Several limitations of the study should be acknowledged. Firstly, we focused here on collecting immediate measures of motivation to connect and did not assess the long-term effects of the interaction. Future studies might explore long-term memories of interactions, along with emotion-related information and real-life behaviors that signify the motivation to connect. Secondly, we solely measured associations between brain activity and behavior. Therefore, it is crucial to investigate causality to determine whether the reported effects genuinely represent the consolidation of social information. Lastly, additional mechanistic investigation is needed to understand how inter-brain coupling supports memory associated with relationships and which specific cues are consolidated.

In conclusion, we demonstrate that post-interaction inter-brain coupling is higher than pre-interaction inter-brain coupling, indicating that between-brain networks are plastic and show durable changes even after the interaction. The findings that post-interaction inter-brain coupling positively predicts motivation to connect may indicate that inter-brain coupling supports the emergence of social relationships. As inter-brain coupling continues after interaction offset and is predictive of subsequent connectedness, it is tempting to speculate that it reflects an early consolidation of social relationships.

## STAR★Methods

### Key resources table

**Table:** 

REAGENT or RESOURCE	SOURCE	IDENTIFIER
**Deposited data**		

Experimental data	This paper	Database: https://data.mendeley.com/datasets/xd9zr7hh3z/1 https://doi.org/10.17632/xd9zr7hh3z.1

**Software and algorithms**		

Analysis scripts	This paper	Database: https://data.mendeley.com/datasets/xd9zr7hh3z/1 https://doi.org/10.17632/xd9zr7hh3z.1

### Resource availability

#### Lead contact

Further information and requests for resources should be directed to and will be fulfilled by the lead contact, Simone Shamay-Tsoory (sshamay@psy.haifa.ac.il).

#### Materials availability

This study did not generate reagents.

#### Data and code availability

• Data: The datasets and analyses code from the current study have been deposited in a public repository. https://doi.org/10.17632/xd9zr7hh3z.1.
• Code: fNIRS preprocessing was performed using HOMER2 software, available online (https://support.razer.com/console/razer-hydra/). Subsequent WTC calculations and statistical analyses were performed using custom code, available in a Mendeley public repository. https://doi.org/10.17632/xd9zr7hh3z.1.
• The lead contact will provide any additional information needed to reanalyse the data reported in the paper.

### Experimental model and study participant details

#### Participants

Eighty participants were recruited through advertisements posted in social media at the University of Haifa. All participants had normal vision and no history of psychiatric or neurological disorders. Participants were between 19 and 28 years old, including 16 males and 64 females. As development of relationship was critical for the experiment, we ensured that participants were not familiar with each other. All participants were randomly assigned into same-sex dyads. Eight dyads were excluded due to data acquisition difficulties. Therefore, the final sample consisted of 64 participants. Participants were unaware of the experimental hypothesis prior to participation. All participants provided their written informed consent to participate in the study. The Institutional Review Board (IRB) at the University of Haifa approved the experiment (approval number 032/18), including the written consent procedure.

### Method details

#### Face-to-face interaction: The movement synchronization paradigm

To manipulate face-to-face social interaction, we used a paradigm of movement synchronization whereby participants were instructed to hold a 3-D game controller (Razer Hydra controllers^1^) in one hand, and move it in synchrony with each other (right, left, forward, backwards, circles, etc.) for two minutes (behavioral data of 3-dimensional movement synchronization of this sample is reported in Marton-Alper et al.^38^). Baseline measurements of inter-brain coupling were collected immediately prior to the interaction (PRE), and directly following it (POST). During these measurements, the participants were instructed to sit back-to-back and move their respective controllers freely for the duration of one minute. To confirm that the persistent effects of inter-brain coupling do not reflect the coupling observed during the interaction participants had a break of several seconds between blocks. This procedure also allowed the participants to change their positions and sit back-to-back. Furthermore, the configuration change from face-to-face to back-to-back took at least 30 seconds.

#### Measure of self-report connectedness

As a means of measuring their motivation to connect following the interaction, participants were asked to rate how much they would want to talk with the person the future, on a 7-point Likert scale. The phrasing of the question was as follows: “To what extent do you wish to talk with the interaction partner in the future”.

#### fNIRS data acquisition

Blood oxygenation for each dyad member was measured for the duration of the task using functional Near-Infrared Spectroscopy (fNIRS). Each participant was fitted with a Brite23 fNIRS system from Artinis Medical Systems, Elst., The Netherlands. The system provides a fixed optode placement (montage), consisting of seven transmitting optodes and seven receiving optodes, resulting overall in 23 transmitter-receiver pairs (channels). The transmitting and receiving optodes comprising each channel were spaced ∼35 mm apart. The optodes comprising each channel were set at a distance of 35 mm. Previous studies have shown that this distance producing reliable signals.^60^^,^^61^

An outline of the montage can be seen in Figure 2. Each channel was configured to operate at wavelengths of 760 and 850 nm, allowing for measurement of concentrations of oxygenated (O_2_Hb) and deoxygenated (HHb) hemoglobin within the corresponding external cortical areas, at a rate of 10 samples/second. Data was collected and analyzed using Atrinis Medical Systems’ OxySoft software, version 3.0.52.

#### fNIRS data analysis

fNIRS Data collected from the participants during the task were pre-processed using the HOMER2^39^ package for the Matlab programming language. For each participant, raw Optical Density (OD) values were imported from OxySoft into the HOMER2 package. Motion artifact corrections were applied to the raw OD values, according to Molavi & Dumont (62). Following this, the corrected OD values were O_2_Hb and HHb concentration values using the modified Beer–Lambert law and applying partial volume correction using Differential Path-length Factor (DPF).^62^ Following the findings of Hoshi,^40^ we chose the O_2_Hb values for further analyses, as more representative of cerebral blood flow, whereas HHb values were discarded as being more likely to be affected by venous blood oxygenation and volume in the intervening tissues. O_2_Hb concentration time series from each channel underwent a visual inspection in the HOMER2 graphical interface for signs of excessive noise and other types of interference. Channels that exhibited excessive noise following the motion artifact removal, or failed to exhibit a pattern typical of heartbeat were excluded entirely from the analyses.

To compensate for artifacts related to scalp blood flow, due to lack of Short Separation Channels in the Brite23 system, we used Principle Component Analysis (PCA)-based spatial filtering, as proposed by Zhang, Noah & Hirsch.^63^ By this method, the O_2_Hb signal was decomposed using PCA. Spatial smoothing was applied to the resulting components, based on known optode positions. These smoothed components were subtracted from the original PCA components, and the O_2_Hb signal was reconstructed using the differences between the original and the smoothed components, thus removing global effects, such as scalp blood flow. This approach allows effectively capturing information related to significant signal variations originating from deep-tissue activation, as per our adopted methodology. Mayer wave can be presumed to represent one such common component, given its origin in arterial blood flow fluctuations, as demonstrated by studies such as Yücel et al*.*^64^ Consequently, we anticipated that this wave would impact all brain regions relatively uniformly. Thus, our utilization of the PCA component subtraction technique was designed to eliminate signal components linked to the Mayer wave’s influence.

#### Inter-brain coupling

Coupling between regions within and between brains was estimated using a Wavelet Transform (WTC) function^41^ in the WTC-16 toolbox for the MATLAB programming language,^65^ which allowed us to identify locally phased-loop activity matching an algorithmically defined wavelet function (a "mother” wavelet). We used a Morlet wavelet functions in the frequency range of 0.015 to 0.15 Hz as the mother wavelet. This excluded all artifacts related to breathing (typically, ∼0.2–0.3 Hz) and heartrate (typically, ∼1–2 Hz). The overall timeseries of the experiment were divided by experiment blocks: Pre-interaction back-to-back baseline (Pre), Interaction (INT), and Post-interaction back-to-back baseline (Post). Per-channel coherence values were obtained by averaging the results of the WTC function across the defined Morlet frequency range and across time for each block. The distributions of these coherence averages were normalized by applying a Fisher’s Z transformation.^66^ The normalized channel averages were then assigned to the following anatomical regions according to each channel’s position on the scalp: Right Inferior Frontal Gyrus (rIFG), Left Inferior Frontal Gyrus (lIFG), and Dorso-Medial Prefrontal Cortex (dmPFC). These areas were selected in accordance with the brain areas known to be associated with social cognition.^67^ We used Linear Mixed Effects (LME) models using the R language,^42^ and the *lme4* package for the R language.^43^ In all instances, inter-brain coupling values which exceeded ±2.5 SD from the general mean were excluded (8.46%). Two types of pairings of brain areas were used in the current work: *Real pairings*, consisting of data from participants who physically performed the task as a dyad, and *Pseudo-pairings,* which were constructed by matching data from participants taken at random from different dyads.

### Quantification and statistical analysis

We initially examined the difference between the brain activity collected from the dyads in our experiment and chance level activation by constructing 2919 pseudo-dyads by means of coupling brain activation from participants who were not members of the same dyad. We then verified that the mean WTC values from the real dyads were outside (above) the 95% Confidence Interval (CI) of the pseudo-dyads’ WTC value distribution for each of the experimental condition.

For the rest of the analyses, we used Linear Mixed Effects (LME) models using the R language,^42^ and the *lme4* package for the R language.^43^ To examine whether participants exhibit differences in inter-brain coupling between the various task conditions, we constructed two LME models, which included Condition (PRE, INT, POST), and ROI pairings as fixed factors, dyads’ ID numbers as a random factor, and coherence (WTC) values as a dependent measure.

To examine brain and behavior relationships we constructed three LME models, each consisting of Condition, ROI, and the continuous value of WTC as fixed factors and dyad IDs as a random factor, for prediction of levels of motivation to connect.
